# Triptolide Downregulates the Expression of NRF2 Target Genes by Increasing Cytoplasmic Localization of NRF2 in A549 Cells

**DOI:** 10.3389/fphar.2021.680167

**Published:** 2021-09-08

**Authors:** Le Ba Nam, Won Jun Choi, Young-Sam Keum

**Affiliations:** ^1^ College of Pharmacy and Integrated Research Institute for Drug Development, Dongguk University, Goyang, South Korea; ^2^ Panacea Co., Goyang, South Korea

**Keywords:** triptolide, NF-E2-related factor 2 (Nrf2), chromosomal maintenance 1 (CRM1), leptomycin B (LMB), nuclear export signal (NES)

## Abstract

We have identified triptolide as a novel NRF2 inhibitor, which significantly attenuates ARE-luciferase activity at nanomolar concentrations. Triptolide did not affect the level of NRF2, but significantly inhibited the expression of NRF2 target genes in A549 cells. We found that NRF2 possesses a previously unrecognized NES in the Neh2 domain, and that triptolide promotes an interaction between NRF2 and CRM1. Triptolide also decreased nuclear accumulation of NRF2, suggesting that it promotes nuclear export of NRF2. In addition, we show that triptolide decreased the expression of NRF2 target genes and increased intracellular oxidative stress, suppressing invasion and promoting cisplatin-induced apoptosis in A549 cells. Finally, oral administration of triptolide suppressed the growth of A549 xenografts in athymic mice by decreasing the expression of NRF2 target genes and promoting oxidative damages via the nuclear export of NRF2 and CRM1 *in vivo*. To the best of our knowledge, triptolide is the first type of compound to inhibit NRF2 by increasing cytoplasmic localization of NRF2.

## Introduction

NF-E2-related factor 2 (NRF2) is responsible for transcriptional activation of phase II cytoprotective enzymes by binding to the antioxidant response element (ARE), a *cis*-acting motif that exists in the promoter of NRF2 target genes (Itoh et al., 1997). The stability of NRF2 is controlled by Kelch-like ECH-associated protein 1 (KEAP1), an adaptor for Cullin 3 (CUL3)-based E3 ubiquitin ligase under basal conditions: CUL3/KEAP1 E3 ubiquitin ligase promotes poly-ubiquitination of NRF2 in the cytosol (Kobayashi et al., 2004). Oxidants and electrophiles halts poly-ubiquitination of NRF2 by inactivating KEAP1, which allows NRF2 to rapidly accumulate, translocate to the nucleus, and activate transcription of NRF2 target genes by forming a heterodimer with small musculoaponeurotic fibrosarcomas (MAFs) (Taguchi et al., 2011). While NRF2 activators provide beneficial therapeutic effects in a variety of stress-related diseases (Cuadrado et al., 2019), aberrant NRF2 activation is often observed in many types of tumors and is closely correlated with poor prognosis because NRF2 activation confers significant advantages towards the growth, proliferation, and metastasis of cancer (Rojo De La Vega et al., 2018).

Cancer genome sequencing studies have identified frequent gain of function mutations in the KEAP1/NRF2 pathway in human non-small cell lung carcinoma (NSCLC) (La Fleur et al., 2019). Specifically, the KEAP1/NRF2 pathway is significantly altered in human lung squamous carcinoma (LUSC) accounting for 34% of genomic alterations, such as somatic mutations and copy number variations (Cancer Genome Atlas Research Network, 2012). Similarly, 23% of human lung adenocarcinoma (LUAD) harbors genomic alterations in the KEAP1/NRF2 pathway (Cancer Genome Atlas Research Network, 2014). While Keap1 mutations are widely distributed, a unique feature of Nrf2 mutations is that they are exclusively clustered in the KEAP1 binding motifs (Taguchi et al., 2011). In addition, the exon 2 in the Nrf2 locus is recurrently lost via alternative splicing and this contributes to the production of NRF2 isoform lacking the Neh2 domain, suggesting an alternative mechanism how NRF2 is activated in lung cancer cells that do not bear mutations in the KEAP1/NRF2 pathway (Goldstein et al., 2016). Taken together, these studies provide evidence that the release of NRF2 from KEAP1 is important for the survival and the proliferation of lung cancer (Taguchi and Yamamoto, 2017).

Considering the abundance of lung tumors exhibiting NRF2 activation and the limited number of available NRF2 inhibitors, the development of NRF2 inhibitors is of a great therapeutic advantage (Jung et al., 2018). Because the structural information of NRF2 is lacking, however, it is impossible to perform the *in silico* analysis of small molecules that might dock to the relevant domains of NRF2. Therefore, scientists have performed the ARE-luciferase assay to identify new NRF2 inhibitors. After discovering brusatol as the first NRF2 inhibitor (Ren et al., 2011), subsequent studies have reported various NRF2 inhibitors with different chemical structures (Robledinos-Anton et al., 2019). Our group identified the Na^+^/K^+^-ATPase inhibitors (Nam and Keum, 2020) and homoharringtonine (Kang et al., 2019) as novel NRF2 inhibitors, suggesting that the Na^+^/K^+^-ATPase and the G-quadruplex structure in the Nrf2 mRNA can be targeted for the development of NRF2 inhibitors (Kang et al., 2020). In an attempt to further develop NRF2 inhibitors, we have observed that triptolide inhibits NRF2 with a distinct mechanism of action compared with other NRF2 inhibitors: triptolide does not affect the level of NRF2, but suppresses the expression of NRF2 target genes by facilitating cytoplasmic localization of NRF2 in A549 cells. In addition, we have identified a novel nuclear export signal (NES) existing in the Neh2 domain of NRF2.

## Materials and Methods

### Cell Culture, Chemicals, and Antibodies

Human lung adenocarcinoma A549 cells, human non-small cell lung carcinoma H1299 cells and human embryonic kidney 293Tcells were cultured in Dulbecco’s Modified Eagle Medium (DMEM) supplemented with 10% fetal bovine serum (FBS) and 1% penicillin/streptomycin (Pen/Strep). DMEM and FBS were purchased from GenDEPOT (Austin, TX, United States). Phosphate-buffered saline (PBS) and Pen/Strep were purchased from WELGENE (Daegu, Korea). Triptolide and cisplatin were obtained from Tokyo Chemical Industry (Tokyo, Japan). The natural compound library was purchased from MedChemExpress (South Brunswick, NJ, United States). FLAG antibody (M2), FLAG-HRP, anti-FLAG beads, anti-HA beads, paraformaldehyde and bovine serum albumin (BSA) were purchased from Sigma-Aldrich (St. Louis, MO, United States). Leptomycin B (LMB) was purchased from Santa Cruz Biotechnology (Santa Cruz, CA, United States). The antibody against HO-1 was purchased from Enzo Life Science (Farmingdale, NY, United States). Antibodies against NQO1 and 4-HNE were purchased from Abcam (Cambridge, MA, United States). Antibodies against actin, CRM1, and 8-OHdG were purchased from Santa Cruz Biotechnology (Santa Cruz, CA, United States). Antibodies against NRF2, cleaved poly (ADP-ribose) polymerase (PARP), cleaved Caspase-3, and HA-HRP were purchased from Cell Signaling Technology (Danvers, MA, United States). JetPEI was purchased from Polyplus transfection (New York, NY, United States).

### Transfection of siRNAs

Transfection of siRNAs was performed according to the manufacturer’s instructions. When cells reached approximately 70% confluence, they were transfected with siRNAs using JetPEI, allowed to grow for additional 48 h, and harvested for biochemical analyses. The Crm1 siRNA sequences are as follows: Sense: CUC​UCU​GAA​GUG​CCU​CAC​U; Antisense: AGU​GAG​GCA​CUU​CAG​AGA​G. The Nrf2 siRNA sequences are as follows: Sense: GAG​ACU​ACC​AUG​GUU​CCA​A; Antisense: UUG​GAA​CCA​UGG​UAG​UCU​C.

### Firefly Luciferase Assay

A549-ARE-GFP-luciferase and H1299-ARE-GFP-luciferase cells were previously established in our laboratory (Lee et al., 2018b). After treatment, the cells were washed three times with ice-cold 1x PBS and lysed with luciferase lysis buffer (100 mM potassium phosphate buffer at pH 7.8, 1% Triton X-100, 1 mM DTT, and 2 mM EDTA) for 1 h. The cell lysates were collected by centrifugation and the luciferase activity was monitored using the GLOMAX Multi-system (Promega, Madison, WI, United States) followed by normalization of the protein concentration.

### MTT Assay

The cells were seeded in 96-well culture plates. After treatment, the cells were washed three times with ice-cold 1x PBS and incubated in a mixture of 180 μl DMEM and 20 μl MTT solution (500 μg/ml) for 4 h. The cells were lysed with 100 μl DMSO for 30 min, and absorbance was measured by spectrophotometer at a wavelength of 560 nm.

### Trypan Blue Exclusion Assay

The cells were plated in quadruplicate in 6-well culture plates and exposed to various concentrations of triptolide for 24 h. The cells were washed with 1x PBS and collected by trypsinization. After washing with 1x PBS, the cells were stained with 0.4% trypan blue solution for 3 min at room temperature. The number of viable cells was counted with a hemocytometer.

### Immunofluorescence

A549 cells were grown on a slice glass and incubated with blocking serum (1% BSA) for 30 min. After washing with 1× PBS, the cells were fixed in paraformaldehyde and hybridized with primary antibodies overnight at 4 C. The slides were washed with 1× PBS and probed with fluorescein isothiocyanate (FITC)-conjugated anti-rabbit or anti-mouse secondary antibodies (Jackson-ImmunoResearch, West Grove, PA, United States). The fluorescent images were obtained with C2 confocal microscope (Nikon Korea, Seoul, Korea).

### Fractionation of the Nucleus and the Cytosol

Cells were washed with 1x PBS and lysed with cell lysis buffer A (50 mM Tris-HCl, 10 mM NaCl, 5 mM MgCl_2_, and 0.5% NP-40, pH 8.0). The lysates were centrifuged at 13,000 rpm for 10 min and the supernatant was collected as the cytosolic fraction. After washing the remnant pellets twice with cell lysis buffer A, they were resuspended in high salt buffer B (20 mM HEPES, 0.5 M NaCl, 1 mM EDTA, and 1 mM dithiothreitol, pH 7.9) and centrifuged for 15 min after brief sonication. The supernatant was collected as the nuclear fraction. The nuclear and cytosolic fractions were subjected to Western blot analysis after the quantification of protein concentration. Glyceraldehyde 3-phosphate dehydrogenase (GAPDH) and histone H3 (H3) were used as the cytosolic and nuclear fraction markers, respectively. The antibody against GAPDH was purchased from Santa Cruz Biotechnology and the antibody against histone H3 was purchased from Cell Signaling Technology.

### Western Blot Analysis

After treatment, the cells were washed three times with 1x PBS and the cell pellets were collected by centrifugation. The pellets were resuspended in 1x RIPA lysis buffer [50 mM Tris-HCl at pH 8.0, 150 mM NaCl, 1% NP-40, 0.5% deoxycholic acid, 0.1% sodium dodecyl sulfate (SDS), 1 mM Na_3_VO_4_, 1 mM DTT, and 1 mM phenylmethylsulfonyl fluoride (PMSF)] and incubated on ice for 1 h. After cell lysates were collected, the protein concentration was measured using the BCA protein assay kit (Thermo-Fisher Scientific, Waltham, MA, United States). Cell lysates were resolved by SDS-PAGE and transferred to PVDF membranes (Merck-Millipore Korea, Daejeon, Korea). The membranes were incubated in blocking buffer (5% skim milk in 1x PBS-0.1% Tween-20, and 1x PBST) for 1 h and hybridized with appropriate primary antibodies in 1x PBS overnight at 4°C. After washing three times with 1x PBST for 30 min, the membrane was hybridized with horseradish peroxidase (HRP)-conjugated secondary antibody (Thermo-Fischer Scientific) for 1 h at 4 C. The membranes were washed three times with 1x PBST for 30 min and visualized using an enhanced chemiluminescence (ECL) detection system.

### Real-Time Reverse Transcription-Polymerase Chain Reaction

Total RNA was extracted with the Hybrid-R RNA extraction kit (GeneAll, Seoul, Korea). Total RNA was subjected to reverse transcription and PCR amplification, using the PrimeScript RT-PCR kit (TAKARA Korea, Seoul, Korea). The real-time RT-PCR analysis was performed using SYBR Mix on a CFX384 Real-time System as recommended by the manufacturer (BioRad Laboratories, Hercules, CA, United States). The mRNA levels of individual genes were normalized by that of GAPDH. The real-time RT-PCR primer sequences are listed in Table 1.

**TABLE 1:** Real-time RT-PCR primers

Genes	Forward	Reverse
Nrf2	CGG​TAT​GCA​ACA​GGA​CAT​TG	ACT​GGT​TGG​GGT​CTT​CTG​TG
Ho-1	GGG​AAT​TCT​CTT​GGC​TGG​CT	CAC​GCA​TGG​CTC​AAA​AAC​CA
Nqo1	GGT​TTG​GAG​TCC​CTG​CCA​TT	GCC​TTC​TTA​CTC​CGG​AAG​GG
Gapdh	CCA​TGG​GGA​AGG​TGA​AGG​TC	TGA​TGA​CCC​TTT​TGG​CTC​CC

### Generation of Stable Cells

Stable A549 cells were generated by transfection of pcDNA3-puro-HA-NRF2 or pcDNA3-HA-NRF2 mutant plasmids, followed by selection of transfected cells with puromycin selection (1 μg/ml) for 48 h. Human Nrf2 and Crm1 cDNAs were amplified from 293T cells using RT-PCR and ligated into pcDNA3-HA-puro and pcDNA3-FLAG-puro vectors. Mutant Nrf2 plasmids were created by overlapping PCR. The sequences of the plasmids were confirmed by DNA sequencing.

### Invasion Assay

Invasion assay was conducted using the Costar Transwell System (Corning Inc., Corning, NY, United States), which bears an 8.0 µm pore size membrane in a plastic ware. The Watrigel Matrix (Corning Inc., Corning, NY, United States) was laid on the membrane in the upper chamber for 12 h. A549 cells were seeded at a density of 5 × 10^4^ cells/well with serum-free DMEM into the upper chamber in the absence or presence of triptolide. After 24 h, cells that invaded in the lower chamber were fixed in 3.7% formaldehyde for 2 min, permeabilized with methanol for 10 min, and stained with Mayer’s Hematoxylin for 15 min. The number of invaded cells was counted in selected random fields of wells using the Eclipse Ti-U inverted microscope (Nikon, Tokyo, Japan).

### A549 Xenograft Study

Six-week old Balb/c nude mice were purchased from Daehan Biolink Co. (Eumseong, Korea). After 1 week acclimation, the mice were subcutaneously injected with A549 cells (5 × 10^6^ cells/mouse) into right the dorsal flank. After 1 week, the mice were orally administered with triptolide everyday (10, 20, and 30 nmol/mouse/day). The weight of the mice and the sizes of the tumors were measured every 3 days during the experiment. The mice were sacrificed by asphyxiation with CO_2_, and the tumors were excised and weighed. The tumor samples were stored either for biochemical analyses or for immunohistochemistry. The animal experiment was performed under the Institutional Animal Care and Use Committee-approved Protocol (IACUC-2020-026-1) of Dongguk University (Seoul, Korea).

### Immunohistochemistry

Tissue samples were fixed in formalin solution at room temperature. The fixed samples were embedded in paraffin and sectioned at 8 μm with the microtome. The tissue slices were mounted on slides and deparaffinized with xylene, followed by multiple hydration steps with increasing concentrations of ethanol. Citrate buffer (pH 6.0) was used for antigen retrieval, and the slides were heated in a microwave for 15 min. The slides were blocked with the IHC blocking solution (ScyTek, Logan, Utah, United States) and incubated with primary antibodies. After hybridization with primary antibodies, the slides were washed multiple times with 1x PBS and incubated with UltraTEk anti-rabbit or anti-mouse HRP-conjugated secondary antibodies (ScyTek, Logan, Utah, United States). The slides were developed with the DAB Kit (GBI Labs, Mukilteo, WA, United States) and counterstained with hematoxylin and eosin. Alternatively, the slides were washed with 1× PBS after hybridization with primary antibodies and probed with FITC-conjugated anti-rabbit or anti-mouse secondary antibodies (Jackson-ImmunoResearch, West Grove, PA, United States). The fluorescent images were obtained with the C2 confocal microscope (Nikon Korea, Seoul, Korea).

### Statistical Analysis

The statistical analysis was conducted using one-way analysis of variance (ANOVA). The asterisks indicate a statistical significance: *p < 0.05, **p < 0.01, and ***p < 0.01.

## Results

### Triptolide Inhibits NRF2 Target Genes by Increasing Cytoplasmic Localization of NRF2 in A549 Cells

**FIGURE 1:**
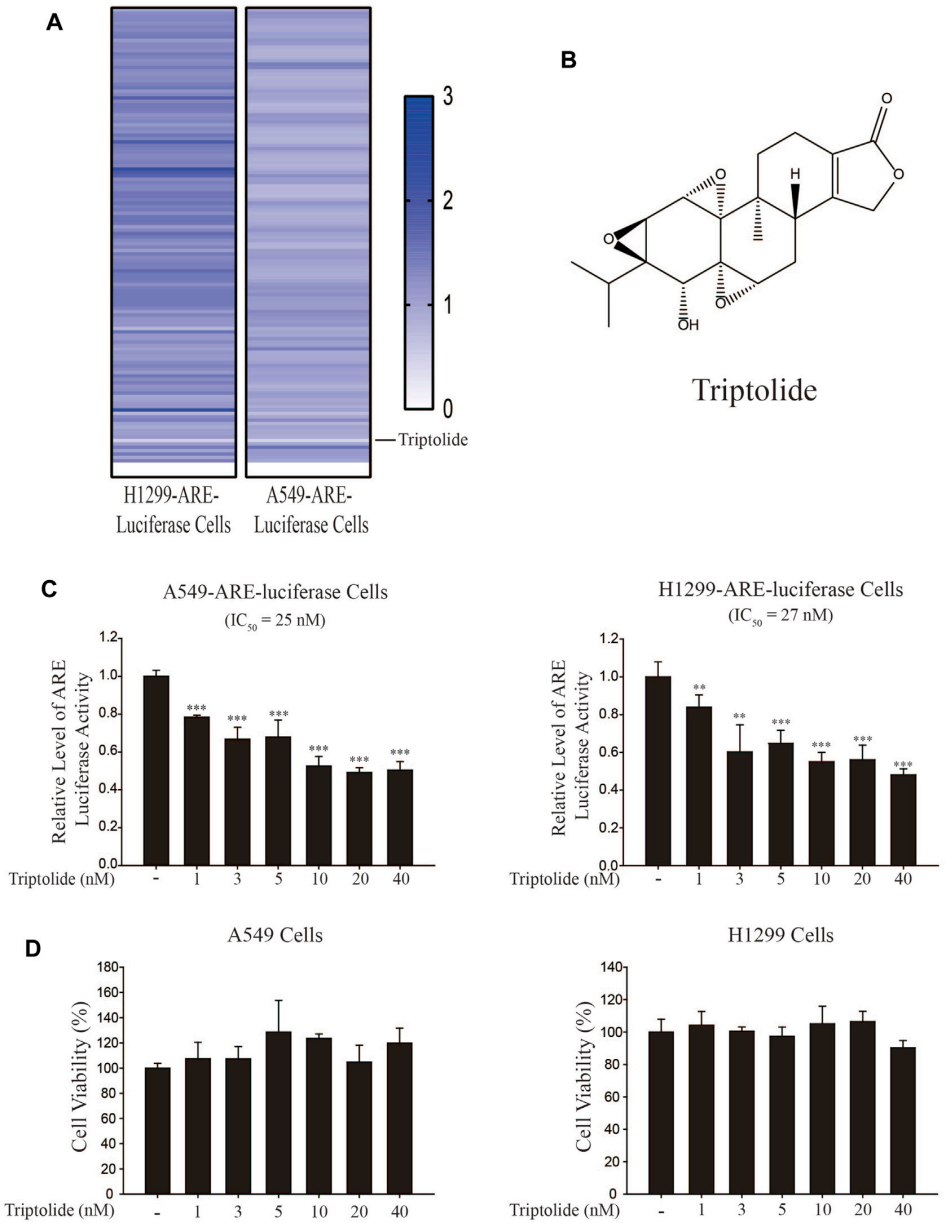
Identification of triptolide as an NRF2 inhibitor. **(A)** Experimental setup for NRF2 inhibitor screening. Triptolide was identified as the strongest ARE-luciferase inhibitor in H1299-ARE-luciferase and A549-ARE-luciferase cells. **(B)** Chemical structure of triptolide. **(C)** Triptolide inhibits ARE-luciferase activity in A549-ARE-luciferase cells (Left Panel) and H1299-ARE-luciferase cells (Right Panel) in a dose-dependent manner. A549-ARE-GFP-luciferase and H1299-ARE-GFP-luciferase cells were seeded in 24-well culture plates (2 × 10^5^ cells/well) and exposed to triptolide at multiple concentrations for 24 h. Cells were collected and the luciferase activity was measured. Asterisks indicate a statistical significance (*n* = 3): **p < 0.01 and ***p < 0.001. **(D)** Triptolide does not affect the viability of A549 cells (Left Panel) and H1299 cells (Right Panel) at concentrations up to 40 nM. A549 and H1299 cells were seeded in 96-well culture plates (4 × 10^4^ cells/well) and MTT assay was conducted (*n* = 3) after triptolide was added for 24 h.

**FIGURE 2:**
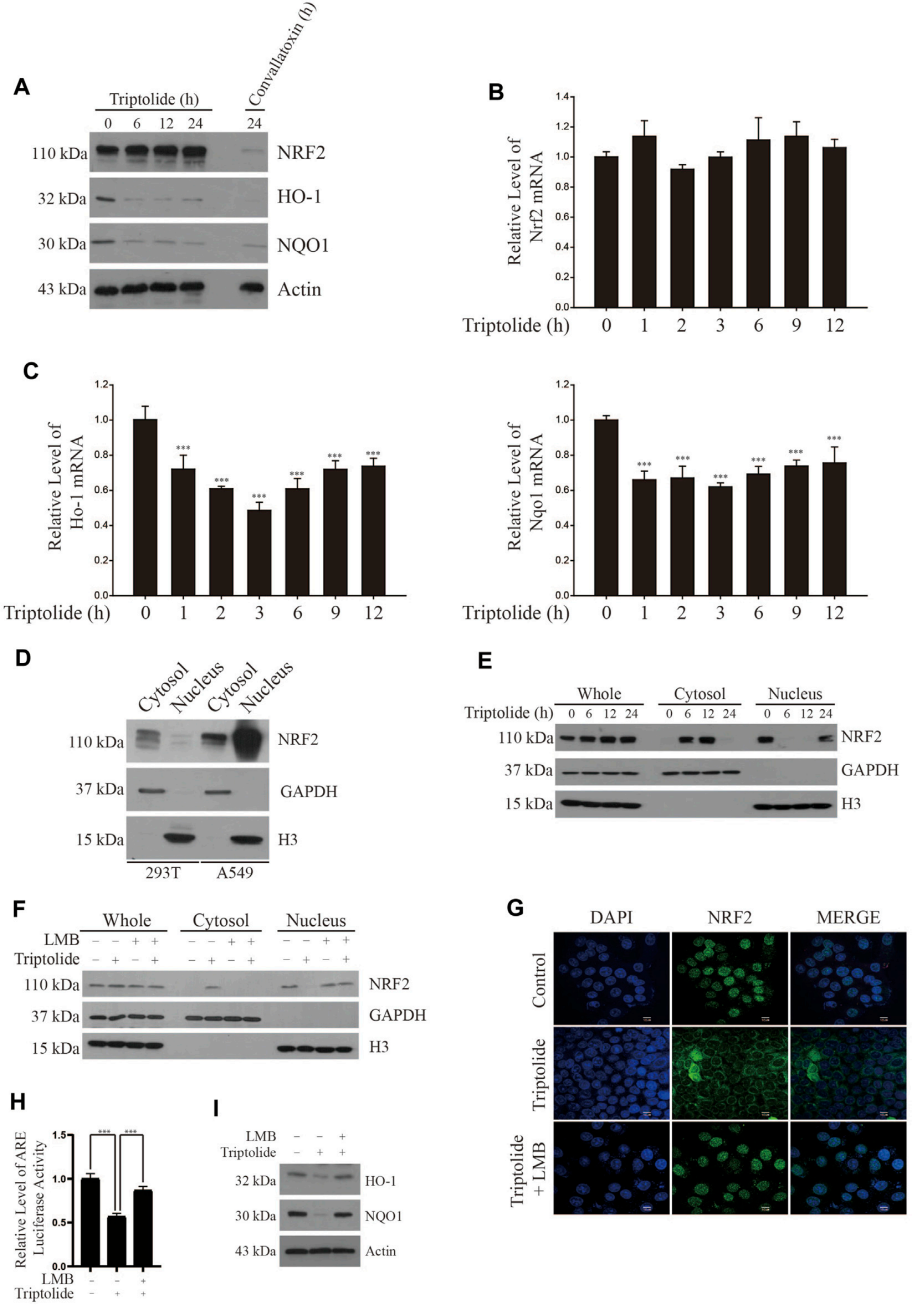
Triptolide increases cytoplasmic localization of NRF2 in A549 cells. **(A)** Triptolide does not affect the level of NRF2, but suppress the expression of HO-1 and NQO1 in A549 cells. Convallatoxin was included as a positive control. A549 cells were seeded in 100 mm culture plates (2 × 10^6^ cells) and exposed to triptolide (20 nM) for various amounts of time. Western blot analysis was performed against NRF2, HO-1, NQO1, and actin **(B)** Triptolide does not affect the level of Nrf2 mRNA in A549 cells. A549 cells were seeded in 6-well culture plates (2.0 × 10^5^ cells/well) and exposed to triptolide (20 nM) for various amounts of time. Real-time RT-PCR was performed against Nrf2 mRNA (*n* = 4). **(C)** Triptolide significantly inhibits the mRNA levels of Ho-1 (Left Panel) and Nqo1 (Right Panel) in A549 cells. A549 cells were seeded in 6-well culture plates (2.0 × 10^5^ cells/well) and exposed to triptolide (20 nM) for various amounts of time. Real-time RT-PCR was performed against Ho-1 and Nqo1 mRNAs. Asterisks indicate a statistical significance (*n* = 4): *p < 0.05 and ***p < 0.001. **(D)** NRF2 abundantly exists in the nucleus of A549 cells. 293T and A549 cells were seeded in 100 mm culture plates (4 × 10^6^ cells). The cytosol and the nucleus were separated, and they were subjected to Western blot analysis. GAPDH and H3 were used as markers for the cytosol and the nucleus, respectively. **(E)** Triptolide promotes the nuclear exclusion of NRF2 in A549 cells. A549 cells were seeded in 100 mm culture plates (2 × 10^6^ cells/well) and exposed to triptolide (20 nM) for various amounts of time. The cytosol and the nucleus were separated and subjected to Western blot analysis. GAPDH and H3 were used as markers for the cytosol and the nucleus, respectively. **(F)** LMB abrogates the nuclear export of NRF2 by triptolide in A549 cells. A549 cells were seeded in 100 mm culture plates (2 × 10^6^ cells) and exposed to triptolide (20 nM) for 6 h in the absence or presence of LMB (20 ng/ml). Western blot analysis was performed against NRF2. GAPDH and H3 were used as markers for the cytosol and the nucleus, respectively. **(G)** Triptolide promotes the nuclear exclusion of NRF2 in A549 cells. A549 cells were seeded in slice glasses (7.5 × 10^4^ cells/glass) and exposed to triptolide (20 nM) for 6 h in the absence or presence of LMB (20 ng/ml). A549 cells were subjected to immunofluorescence using the NRF2 antibody. The nucleus was stained with DAPI. **(H)** LMB abrogates the inhibition of ARE-luciferase activity by triptolide in A549-ARE-luciferase cells. A549-ARE-GFP-luciferase cells were seeded in 24-well culture plates (2 × 10^5^ cells/well) and exposed to triptolide in the absence or presence of LMB (20 ng/ml) for 24 h. The luciferase assay was performed and asterisks indicate a statistical significance (*n* = 3): ***p < 0.001. **(I)** LMB abrogates the inhibition of NRF2 expression by triptolide in A549 cells. A549 cells were seeded in 100 mm culture plates (2 × 10^6^ cells) and exposed to triptolide (20 nM) in the absence or presence of LMB (20 ng/ml) for 24 h. Western blot analysis was performed against HO-1, NQO1, and actin.

In an attempt to find out a novel NRF2 inhibitor, we have exposed A549-ARE-GFP-luciferase cells and H1299-ARE-GFP-luciferase cells to 10 nM of 2,650 chemicals from a natural compound library for 24 h (Figure 1A). As a result, we found that triptolide (Figure 1B) significantly inhibited ARE-luciferase activity in A549-ARE-GFP-luciferase cells (IC_50_ = 25 nM) and H1299-ARE-GFP-luciferase cells (IC_50_ = 27 nM) (Figure 1C). Concentrations up to 40 nM of triptolide did not affect the metabolic activity of A549 cells and H1299 cells, as assessed by MTT assay (Figure 1D). Trypan blue exclusion assay also indicated that the viability of A549 and H1299 cells was unaffected by triptolide at concentrations up to 40 nM (Supplementary Figure S1). We have previously identified that convallatoxin inhibits NRF2 and sensitizes A549 cells to cisplatin-induced apoptosis (Lee et al., 2018a). Using convallatoxin as a prototypical NRF2 inhibitor, we exposed A549 cells to triptolide, and examined the expression of NRF2 and its target proteins, heme oxygenase-1 (HO-1) and NAD [P]H:quinone oxidoreductase-1 (NQO1) by Western blot analysis. As a result, we observed that triptolide suppressed the expression of HO-1 and NQO1, but it failed to downregulate the expression of NRF2 in A549 cells (Figure 2A). Real-time RT-PCR analysis also indicates that triptolide failed to affect the level of Nrf2 mRNA (Figure 2B), but significantly inhibited the levels of Ho-1 (Left Panel) and Nqo1 (Right Panel) mRNAs in A549 cells (Figure 2C).

A549 cells are derived from an adenocarcinoma of human alveolar basal epithelium and exhibit aberrant activation of NRF2 owing to two mechanisms: one is a somatic mutation in Keap1 at G333C (Singh et al., 2006) and the other is the epigenetic silencing by methylation in the promoter of Keap1 (Wang et al., 2008). To investigate the molecular mechanisms underlying how triptolide inhibits the expression of NRF2 target genes without affecting the level of NRF2, we exposed A549 cells to triptolide and fractionated the nucleus and cytosol. We observed that, unlike 293T cells, NRF2 was abundantly located in the nucleus of A549 cells during basal condition (Figure 2D). Our results also show that triptolide promoted cytoplasmic localization of NRF2 in A549 cells (Figure 2E) and this event was suppressed by cotreatment of leptomycin B (LMB), a nuclear export inhibitor (Fung and Chook, 2014), as examined by Western blot analysis (Figure 2F) and immunofluorescence (Figure 2G). Consistent with these observations, LMB abrogated the inhibition of ARE-luciferase activity (Figure 2H) and that of HO-1 and NQO1 (Figure 2I) by triptolide. Together, these results illustrate that triptolide inhibits the expression of NRF2 target genes by reducing the nuclear accumulation of NRF2 in A549 cells.

### Triptolide Promotes the Nuclear Exclusion of NRF2 by CRM1

**FIGURE 3:**
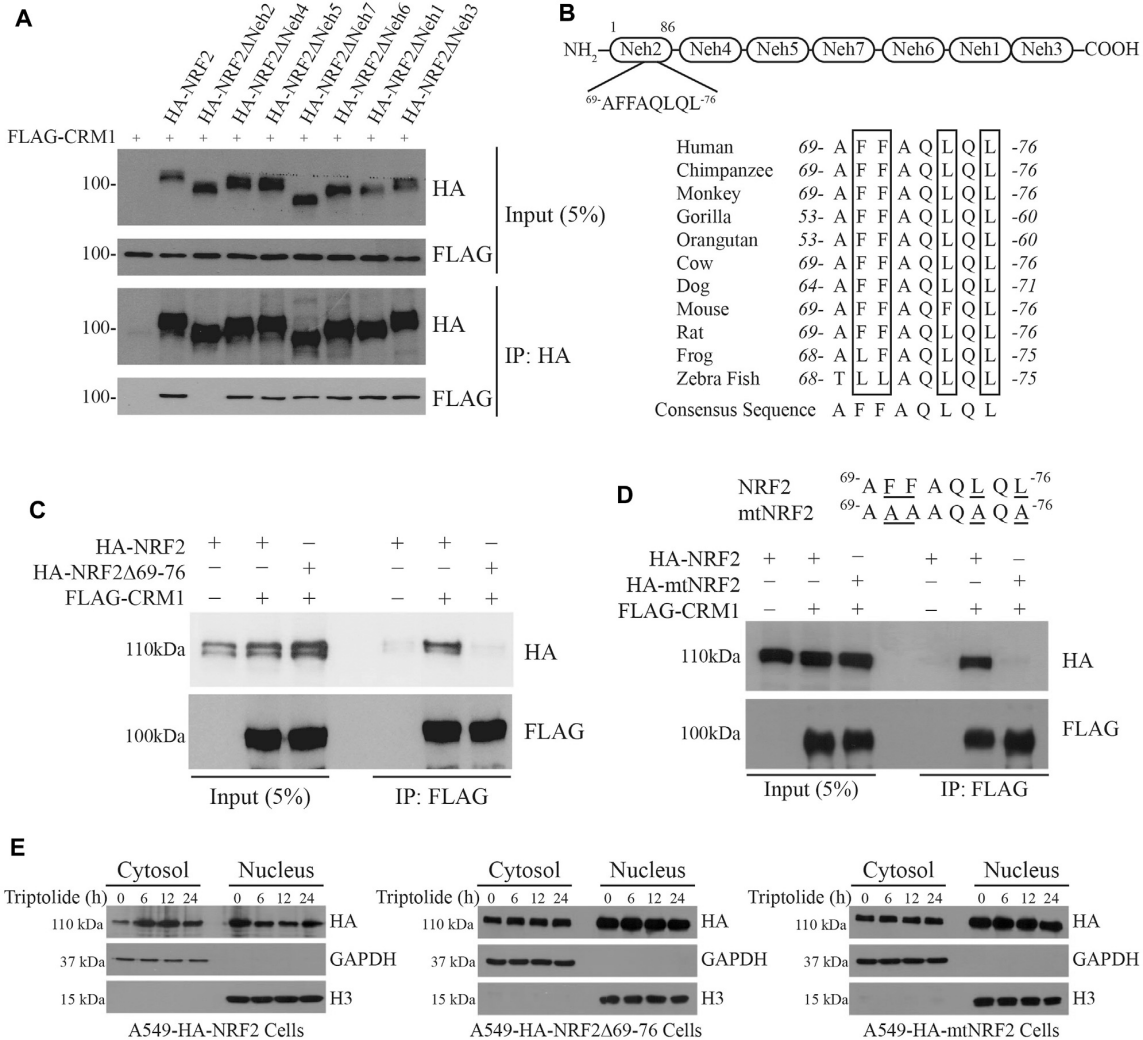
Identification of a novel nuclear export signal (NES) in the Neh2 domain. **(A)** The Neh2 domain is responsible for binding to CRM1. pcDNA3-FLAG-CRM1 plasmid (1 μg) was cotransfected with pcDNA3-HA-NRF2 (1 μg) or pcDNA3-HA-NRF2 plasmids deleted with the individual Neh domains (1 μg) in 293T cells. The cell lysates were collected and immunoprecipitated with anti-HA beads. After immunoprecipitation, Western blot analysis was performed using HA-HRP and FLAG-HRP antibodies. **(B)** Domain architecture of the Neh domains in NRF2 (Upper Panel) and the existence of the NES-like sequence in the Neh2 domain, which is highly preserved across the species (Lower Panel). **(C)** NRF2 deleted with the NES-like sequence in the Neh2 domain fails to bind to CRM1. pcDNA3-FLAG-CRM1 plasmid (1 μg) was cotransfected with pcDNA3-HA-NRF2 (1 μg) or pcDNA3-NRF2Δ69-76 deletion mutant (1 μg) in 293T cells for 24 h. The cells lysates were collected and immunoprecipitated with anti-FLAG beads. After immunoprecipitation, Western blot analysis was performed using FLAG-HRP and HA-HRP antibodies. **(D)** NRF2 mutated in the NES-like sequence in the Neh2 domain (mtNRF2) fails to bind to CRM1. pcDNA3-FLAG-CRM1 plasmid (1 μg) was cotransfected with pcDNA3-HA-NRF2 (1 μg) or pcDNA3-mtNRF2 (1 μg) plasmids in 293T cells for 24 h. The cell lysates were collected and immunoprecipitated with anti-FLAG beads. After immunoprecipitation, Western blot analysis was performed using FLAG-HRP and HA-HRP antibodies. **(E)** Triptolide promotes the nuclear exclusion of HA-NRF2, but not that of NRF2Δ69-76 or HA-mtNRF2. Stable A549-HA-NRF2, A549-HA-NRF2Δ69-76 and A549-HA-mutNRF2 cells were seeded in 100 mm culture plates (2 × 10^6^ cells), and they were exposed to triptolide (20 nM) for various amounts of time. The cytosol and the nucleus were separated, and Western blot analysis was performed against HA. GAPDH and H3 were used as markers for the cytosol and the nucleus, respectively.

Chromosomal Maintenance 1 (CRM1, also known as Exportin 1) is a major mammalian export protein that facilitates the transport of large macromolecules including RNA and proteins across the nuclear membrane into the cytosol (Hutten and Kehlenbach, 2007). While a previous study provided a clue that CRM1 can bind to and regulate the activity of NRF2 (Velichkova and Hasson, 2005), the mode of interaction is still unclear. To address this issue, we cotransfected FLAG-CRM1 plasmid with HA-NRF2 plasmid or with HA-NRF2 plasmids lacking the individual Neh domains in 293T cells and performed immunoprecipitation with HA-agarose beads followed by Western blot analysis: 293T cells were used to monitor the interaction between two epitope-tagged plasmids due to the ease of transfection. Our results show that FLAG-CRM1 failed to bind to HA-NRF2ΔNeh2, suggesting that CRM1 binds to the Neh2 domain of NRF2 (Figure 3A).

A close examination of the amino acid sequence in the Neh2 domain illustrated that the Neh2 domain contains a highly conserved and previously unrecognized nuclear export signal (NES) across the species (Figure 3B). To examine whether this putative NES is critical for the interaction between NRF2 and CRM1, we created the HA-NRF2 plasmid lacking the NES (referred to as HA-NRF2Δ69-76) or a mutant NRF2 plasmid in which all phenylalanine (F) and leucine (L) residues in the putative NES were mutated into alanine (A) (referred to as mtNRF2), and cotransfected them with FLAG-CRM1 in 293T cells followed by immunoprecipitation with HA-agarose beads and Western blot analysis. Our results show that FLAG-CRM1 failed to bind to HA-NRF2Δ69-76 (Figure 3C) and HA-mtNRF2 (Figure 3D), suggesting that this putative NES is critical for the binding of NRF2 to CRM1. To examine whether this putative NES plays a role in the nuclear export of NRF2 by triptolide, we created stable A549 cells overexpressing HA-NRF2, HA-NRF2Δ69-76 and HA-mtNRF2, and exposed them to triptolide. Our results show that triptolide promoted the nuclear export of HA-NRF2, but not that of HA-NRF2Δ69-76 and HA-mtNRF2 (Figure 3E), suggesting that this putative NES is critical for the nuclear export of NRF2 by triptolide.

### The Inhibition of NRF2 Target Genes by Triptolide Is Dependent on CRM1 in A549 Cells

**FIGURE 4:**
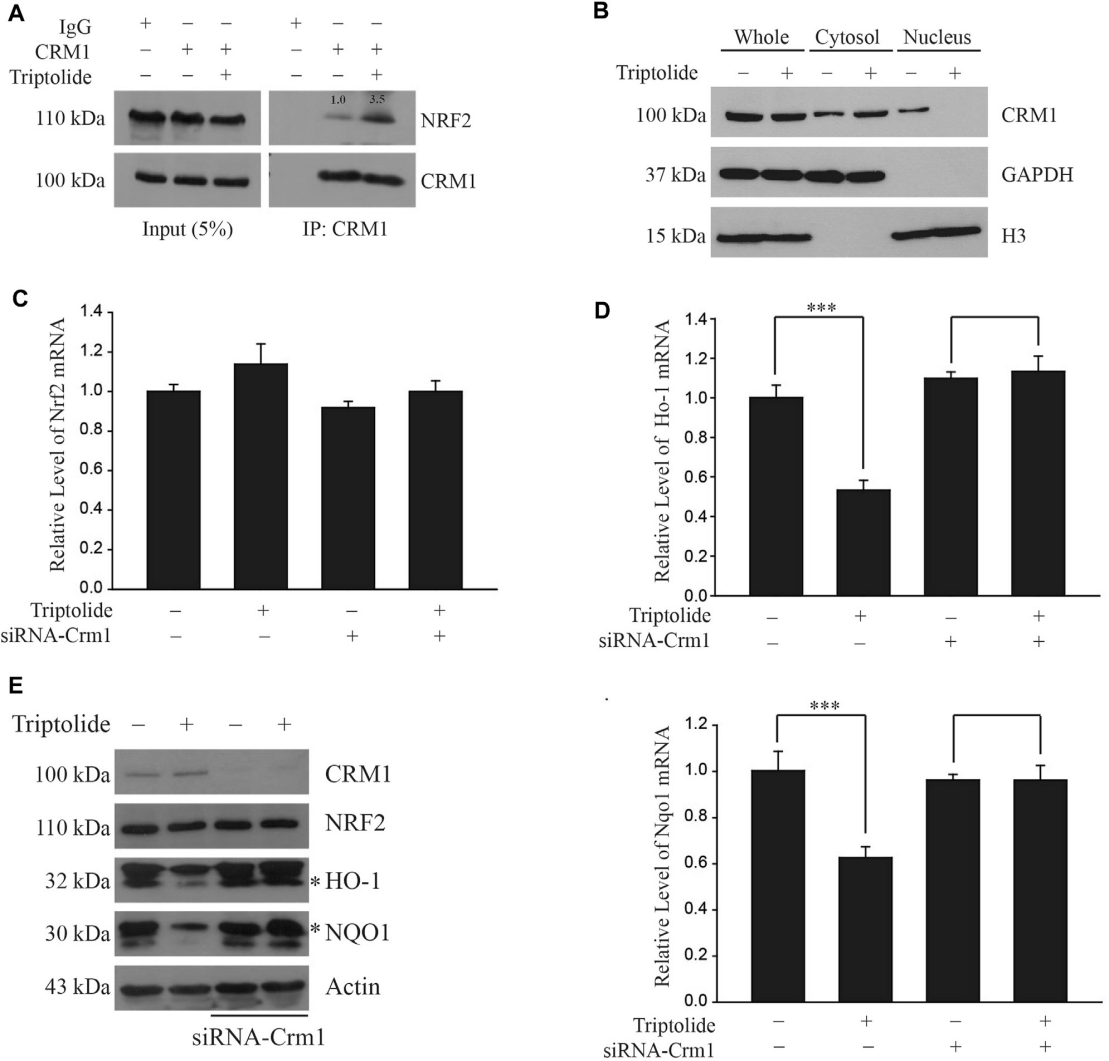
Triptolide promotes the interaction between NRF2 and CRM1 in A549 cells. **(A)** Triptolide promotes the endogenous interaction between NRF2 and CRM1 in A549 cells. A relative density of bands is illustrated inside the figure. A549 cells were seeded in 100 mm culture plates (2 × 10^6^ cells) and exposed to triptolide (20 nM) for 24 h. Cell lysates were collected and incubated with IgG (1 μg) or CRM1 antibody (1 μg) overnight at 4°C. After immunoprecipitation with protein A/G beads, the samples were washed with NP-40 lysis buffer and Western blot analysis was performed against NRF2 and CRM1. **(B)** Triptolide increases cytoplasmic localization of CRM1 in A549 cells. A549 cells were seeded in 100 mm culture plates (2 × 10^6^ cells) and exposed to triptolide (20 nM) for 24 h. After separation of the cytosol and the nucleus, Western blot analysis was performed against CRM1. GAPDH and H3 were used as markers for the cytosol and the nucleus, respectively. **(C)** Triptolide does not affect the level of Nrf2 mRNA regardless of CRM1. A549 cells were seeded in 60 mm plates (2.0 × 10^5^ cells) and exposed to triptolide (20 nM) for 3 h in the absence or presence of siRNA against Crm1 (100 nM). Total RNA was prepared and real-time RT-PCR was performed using Nrf2 ]specific primers (*n* = 4). **(D)** Suppression of transcription of Ho-1 (Left Panel) and Nqo1 (Right Panel) by triptolide is dependent on CRM1. A549 cells were seeded in 60 mm plates (2.0 × 10^5^ cells) and exposed to triptolide (20 nM) for 3 h in the absence or presence of siRNA against Crm1 (100 nM). Total RNA was prepared and real-time RT-PCR was conducted using specific primers against Ho-1 and Nqo1. Asterisks indicate a statistical significance (*n* = 4): ***p < 0.01. **(E)** Suppression of HO-1 and NQO1 by triptolide is dependent on CRM1. A549 cells were seeded in 60 mm plates (2.0 × 10^5^ cells) and exposed to triptolide (20 nM) in the absence or presence of siRNA against Crm1 (100 nM) for 24 h. Cell lysates were collected and Western blot analysis was performed.

We observed that triptolide increased an endogenous interaction between NRF2 and CRM1 in A549 cells (Figure 4A). Triptolide also promoted cytoplasmic localization of CRM1 in A549 cells (Figure 4B). To examine whether the inhibition of NRF2 target genes by triptolide is dependent on CRM1, we knocked down Crm1 in A549 cells and exposed them to triptolide. While triptolide failed to affect the level of Nrf2 mRNA (Figure 4C), knocking down Crm1 abrogated the inhibition of Ho-1 (Upper Panel) and Nqo1 mRNAs (Lower Panel) by triptolide (Figure 4D). Consistent with this observation, knocking-down Crm1 abrogated the inhibition of HO-1 and NQO1 by triptolide (Figure 4E). Together, these results illustrate that the inhibition of NRF2 target genes by triptolide is dependent on CRM1 in A549 cells.

### Triptolide Increases Oxidative Stress, Inhibits Invasion, and Sensitizes Cisplatin-Induced Apoptosis in A549 Cells

**FIGURE 5:**
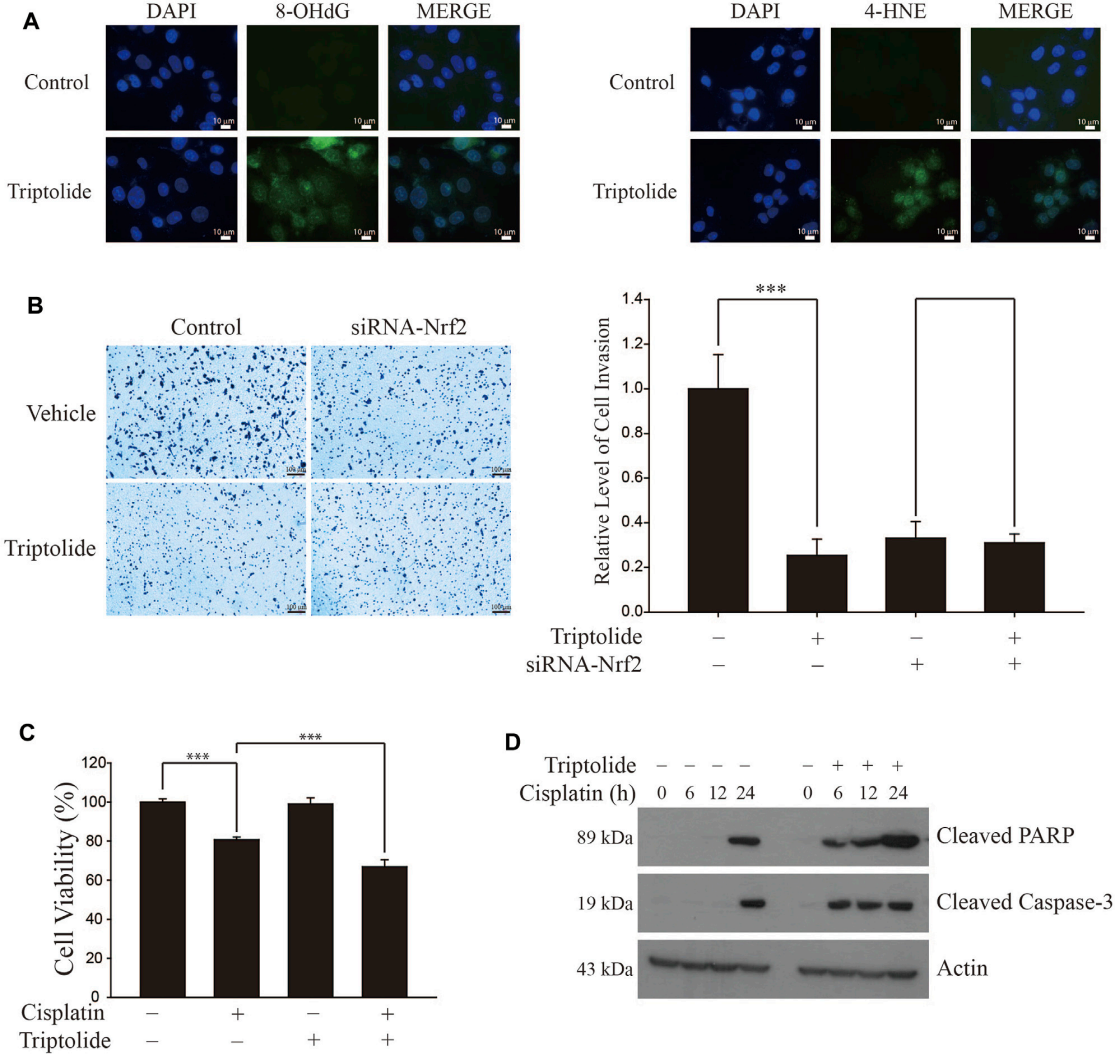
Triptolide induces oxidative stress, inhibits invasion, and sensitizes cisplatin-induced cell death in A549 cells. **(A)** Triptolide induces intracellular oxidative stress. After treatment of triptolide (20 nM) for 24 h, the levels of the oxidative stress markers 8-OHdG (Left Panel) and 4-HNE (Right Panel) were measured by immunofluorescence in A549 cells. **(B)** Triptolide suppresses invasion in A549 cells. The invasion assay was conducted with A549 cells in the absence or presence of triptolide (20 nM) for 24 h. Representative figures are provided (Left Panel) and asterisks indicate a statistical significance with ***p < 0.001 (Right Panel, *n* = 4). **(C)** Triptolide potentiates cisplatin-induced cell death in A549 cells. A549 cells were seeded in 96-well culture plates (4 × 10^4^ cells/well) and exposed to cisplatin (20 µM) in the absence or presence of triptolide (20 nM) for 24 h. MTT assay was conducted to measure the cell viability and asterisks indicate a statistical significance (*n* = 3): ***p < 0.001. **(D)** Triptolide sensitizes cisplatin-induced apoptosis in A549 cells. A549 cells were seeded in 60 mm plates (2.0 × 10^5^ cells/well) and exposed to cisplatin (20 μM) alone or together with triptolide (20 nM). Western blot analysis was conducted using Cleaved PARP, Cleaved Caspase-3, and actin antibodies.

We found that triptolide promoted the generation of intracellular oxidative stress in A549 cells, as illustrated by an increase in the levels of 8-hydroxydeoxyguanine (8-OHdG, Left Panel) and 4-hydroxynonenal (4-HNE, Right Panel) (Figure 5A). Triptolide also inhibited invasion of A549 cells, but this event was attenuated when Nrf2 was silenced by siRNA (Figure 5B). In addition, triptolide potentiated cisplatin-induced cell death (Figure 5C) and increased cisplatin-induced caspase-3 activation, an indicator of apoptosis (Figure 5D). Together, these results illustrate that the inhibition of NRF2 by triptolide is associated with promoting oxidative stress, suppressing invasion, and potentiating cell death and apoptosis in A549 cells.

### Triptolide Inhibits the Growth of A549 Xenografts *In Vivo*

**FIGURE 6:**
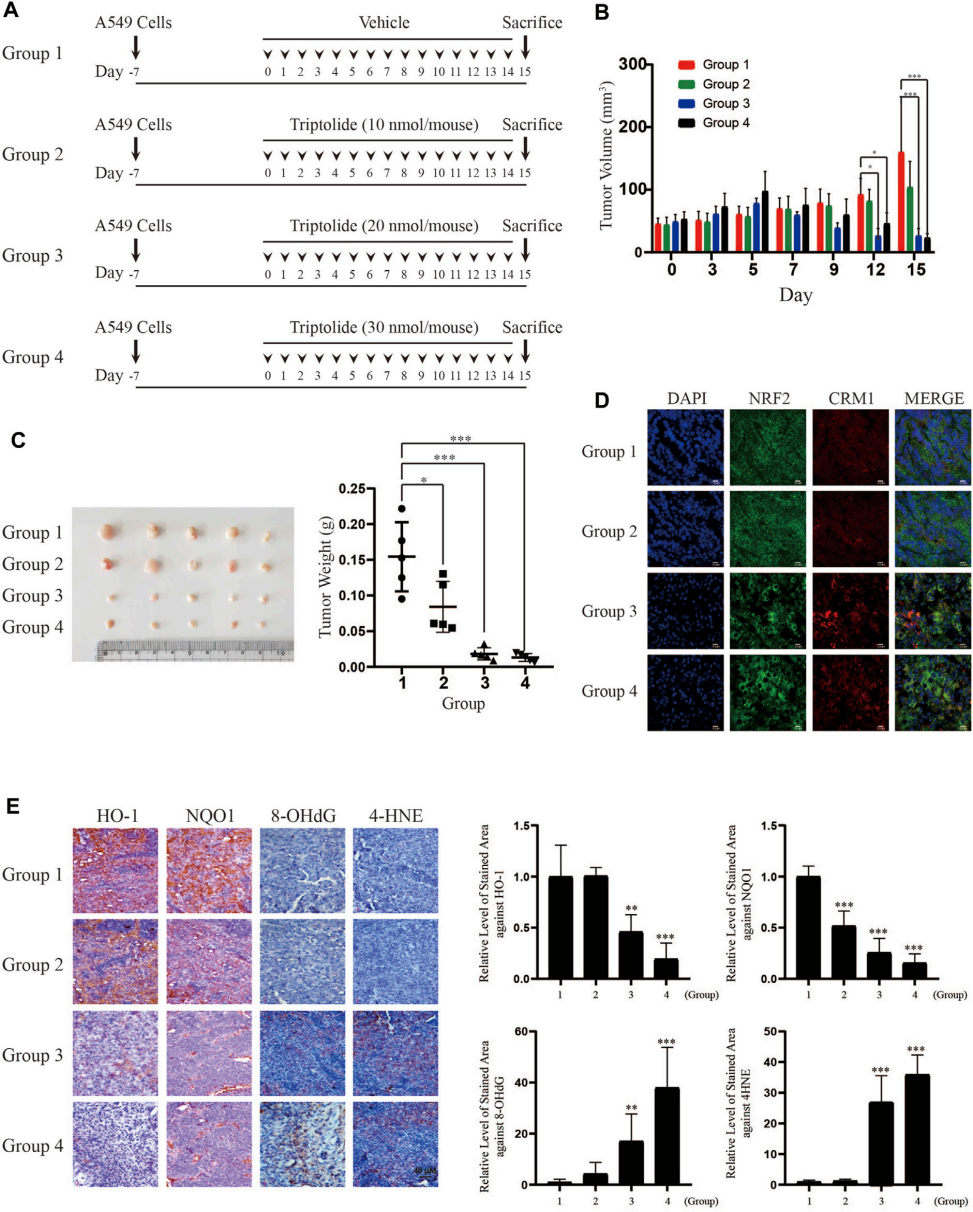
Triptolide inhibits the growth of A549 xenografts in nude mice. **(A)** Experimental scheme of A549 xenograft experiment. **(B)** Triptolide suppresses the growth of A549 xenografts in nude mice. During the experiment, the volume of A549 xenografts was measured by caliper every 3 days and calculated based on the formula, V = LxW^2^/2 (L: Length, W: Width). Asterisks indicate a statistical significance (*n* = 5): *p < 0.05 and ***p < 0.001. **(C)** Triptolide suppresses the weight of A549 xenografts in nude mice. The weight of A549 xenografts was measured at sacrifice. Asterisks indicate a statistical significance (*n* = 5): *p < 0.05 and ***p < 0.001. **(D)** Triptolide promotes nuclear exclusion of NRF2 *in vivo*. Tissue samples of A549 xenografts were subjected to immunofluorescence using NRF2 and CRM1 antibodies. Representative slides of each group are provided. **(E)** Triptolide suppresses the expression of NRF2 target proteins (HO-1 and NQO1) and promotes oxidative stress (8-OHdG and 4-HNE) *in vivo*. The representative images of proteins stained with individual antibodies are provided (Left Panel) and the relative levels of stained area are provided (Right Panel). Asterisks indicate a statistical significance (*n* = 5): **p < 0.01 and ***p < 0.001, compared with the control (Group 1).

In order to examine whether the inhibition of NRF2 by triptolide exerts inhibitory effects on the growth of lung tumors *in vivo*, we injected A549 cells into the flank of athymic nude mice and orally administered them with triptolide (Figure 6A). While triptolide did not affect the body weight of mice during the course of study (**data not shown**), it significantly decreased the growth of A549 xenografts after 12 and 15 days (Figure 6B). At sacrifice, we noticed that triptolide significantly decreased the weight of A549 cell xenografts in a dose-dependent manner (Figure 6C). Immunohistochemistry studies illustrate that triptolide promoted cytoplasmic localization of NRF2 and CRM1 (Figure 6D), inhibited the expression of HO-1 and NQO1 (Figure 6E), and increased the generation of oxidative stress markers (8-OHdG and 4-HNE) in A549 xenografts (Figure 6E). These results illustrate that the inhibition of the growth of A549 xenografts by triptolide is associated with the inhibition of NRF2 target genes, cytoplasmic localization of NRF2 and CRM1, and the generation of oxidative stress *in vivo*.

## Discussion

Triptolide was first isolated from *Tripterygium wilfordii* Hook F, also known as Lei Gong Teng or Thunder God Vine (Kupchan et al., 1972). Thereafter, a number of studies have demonstrated that triptolide is effective against various diseases, including cancer. Because triptolide is a lipophilic compound, its clinical efficacy is limited. Therefore, many attempts to develop triptolide derivatives with higher efficacy, enhanced water solubility, and lower toxicity have been made in the last two decades and two triptolide derivatives (minnelide and F60008) are currently under the clinical trials (Noel et al., 2019). Triptolide is known to affect a number of cellular proteins and intracellular kinase pathways to exhibit anti-carcinogenic effects (Chen et al., 2018). In addition, it is assumed that nuclear hormone receptors could be putative targets for the inhibition of NRF2 by triptolide because triptolide possesses a diterpenoid structure similar to several lipophilic hormones (Liu et al., 2015). However, the mechanistic linkage between the nuclear receptors and NRF2 is currently lacking.

Triptolide has a hydroxyl group deemed important for its anti-tumorigenic activity (Li et al., 2009). Triptolide also possesses four reactive chemical groups that may covalently react with cellular targets: the butenolide moiety in the five-membered lactone ring and the three epoxides. While direct cellular targets for the hydroxyl and the butenolide groups of triptolide are unclear, He *et al.* demonstrated that the epoxide in triptolide targets the XBP1 subunit of transcription factor TFIIH (Titov et al., 2011) and inhibits transcription and nucleotide excision repair activity of RNA polymerase II *via* a covalent modification at Cys342 (He et al., 2015). Zhao *et al.* showed that triptolide targets the peroxiredoxin I (Prx I) and inhibits its chaperone activity, but not peroxidase activity, in an analogous manner: the epoxide in triptolide prevents formation of the Prx I homodecamer via covalent modifications at Cys83 and Cys173 (Zhao et al., 2015). It is unclear whether and, if so, which moieties in triptolide is required for NRF2 inhibition and we are currently conducting the structure-activity relationship (SAR) studies to address this issue. While the roles of the XBP1 subunit and Prx I in the regulation of NRF2 target genes by triptolide is unknown, we observed that knocking down Nrf2 in A549 cells abrogated the inhibition of HO-1 and NQO1 (Supplementary Figure S2A), and that of Ho-1 and Nqo1 mRNAs (Supplementary Figure S2B) by triptolide. In addition, triptolide failed to affect the level of Ho-1 and Nqo1 mRNAs in 293T cells (Supplementary Figure S3). These results suggest that NRF2 plays a major role in the inhibition of NRF2 target genes by triptolide in A549 cells.

In the present study, we have identified that triptolide is a novel NRF2 inhibitor (Figure 1). The unique feature of NRF2 inhibition by triptolide is that, unlike other NRF2 inhibitors, triptolide inhibits the expression of NRF2 target genes without affecting the level of NRF2 (Figure 2). The nuclear export of NRF2 and the inhibition of NRF2 target genes by triptolide was observed *in vitro* (Figure 2) and *in vivo* (Figure 6D). While the detailed molecular mechanisms remain to be further investigated, we observed that triptolide promoted the nuclear exclusion of NRF2 in A549 cells and increased the interaction between NRF2 and CRM1 in whole cell extracts (Figures 3, 4). In addition, we have identified that NRF2 possesses a previously unrecognized NES in the Neh2 domain where CRM1 can bind to and promote the nuclear export of NRF2 (Figure 3). Because the NES in the Neh2 domain lacks potential amino acid residues susceptible to post-translational modifications, it is unlikely that the induction of post-translational modification in the NES promoted the interaction between NRF2 and CRM1 caused by triptolide. We are rather tempted to speculate that triptolide might induce post-translational modification(s) in CRM1 and/or its regulatory proteins to affect cytoplasmic localization of NRF2. Indeed, there exist studies demonstrating post-translational modifications affect the activities of CRM1 (Wu et al., 2013) and its regulatory protein, Ran-GTPase (De Boor et al., 2015). However, due to the lack of appropriate antibodies indicative of post-translational modifications of CRM1 and Ran-GTPase, we could not examine this hypothesis. Collectively, we propose that triptolide has an advantage compared with other NRF2 inhibitors in that it downregulates the expression of NRF2 target genes without affecting NRF2, which could be used to treat lung cancer cells regardless of mutation status in the Keap1/Nrf2 pathway. Understanding the detailed molecular mechanisms how triptolide promotes the nuclear exclusion of NRF2 will provide us a clue to develop NRF2 inhibitors with higher efficacy and lower toxicity.

## Data Availability

The raw data supporting the conclusions of this article will be made available by the authors, without undue reservation.

## Supplementary Material

Supplementray Figure S1 Triptolide does not affect the viability of A549 cells (Left Panel) and H1299 cells (Right Panel) at concentrations up to 40 nM. A549 cells and H1299 cells were seeded in 96-well culture plates (4x10^4^ cells/well) and trypan blue exclusion assay was conducted (*n* = 4) after treatment of triptolide at multiple concentrations for 24 h.

Supplementray Figure S2 Suppression of NRF2 target genes by triptolide requires NRF2 in A549 cells. **(A)** Triptolide fails to suppress the expression of HO-1 and NQO1 in A549 cells when Nrf2 is silenced by siRNA**.** A549 cells were seeded in 100 mm culture plates (2x10^6^ cells) and exposed to triptolide (20 nM) for 24 h in the absence or presence of siRNA against Nrf2 (100 nM). Western blot analysis was performed using NRF2, HO-1, NQO1, and actin antibodies. **(B)** Suppression of Ho-1 (Left Panel) and Nqo1 (Right Panel) mRNAs by triptolide is dependent on NRF2. A549 cells were seeded in 60 mm plates (2.0x10^5^ cells) and exposed to triptolide (20 nM) for 3 h in the absence or presence of siRNA against Nrf2 (100 nM). Total RNA was prepared and real-time RT-PCR was conducted using specific primers against Ho-1 and Nqo1. Asterisks indicate a statistical significance (*n* = 4): ****P*<0.01.

Supplementray Figure S3 Triptolide does not affect the levels of Ho-1 and Nqo1 mRNAs in 293T cells. 293T cells were exposed to triptolide (10 nM) for 3 h. Total RNA of 293T cells and A549 cells was prepared and real-time RT-PCR was conducting specific primers against Ho-1 and Nqo1. Asterisks indicate a statistical significance (*n* = 4): ****P*<0.01.
