# Supplementary figures and images for: Triptolide Downregulates the Expression of NRF2 Target Genes by Increasing Cytoplasmic Localization of NRF2 in A549 Cells
Source: Front Pharmacol. 2021 Sep 8;12:680167. doi: 10.3389/fphar.2021.680167 (PMC8455929; doi:10.3389/fphar.2021.680167)

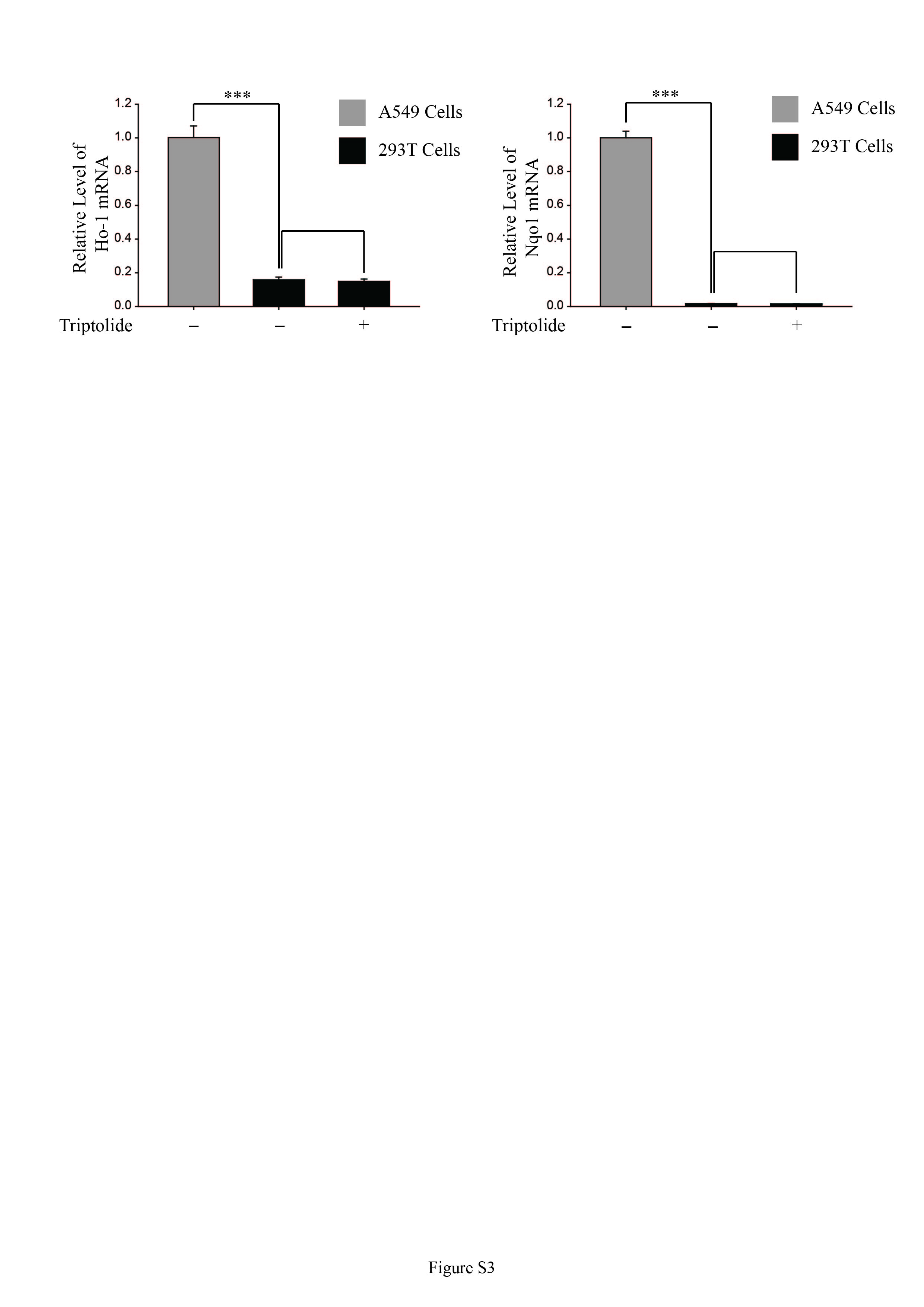

Supplement: Supplementary file 1 [file Image3.JPEG]

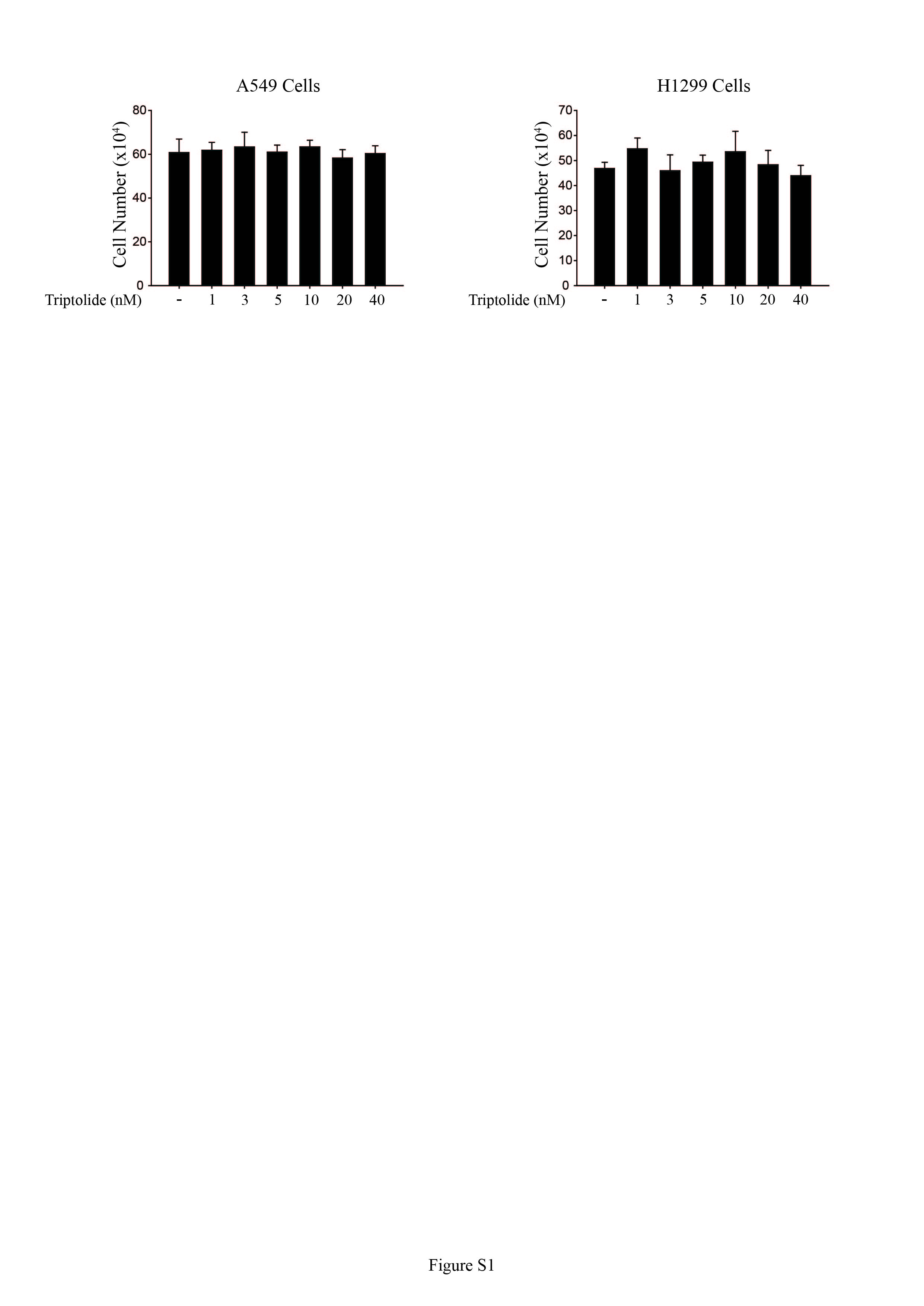

Supplement: Supplementary file 2 [file Image1.JPEG]

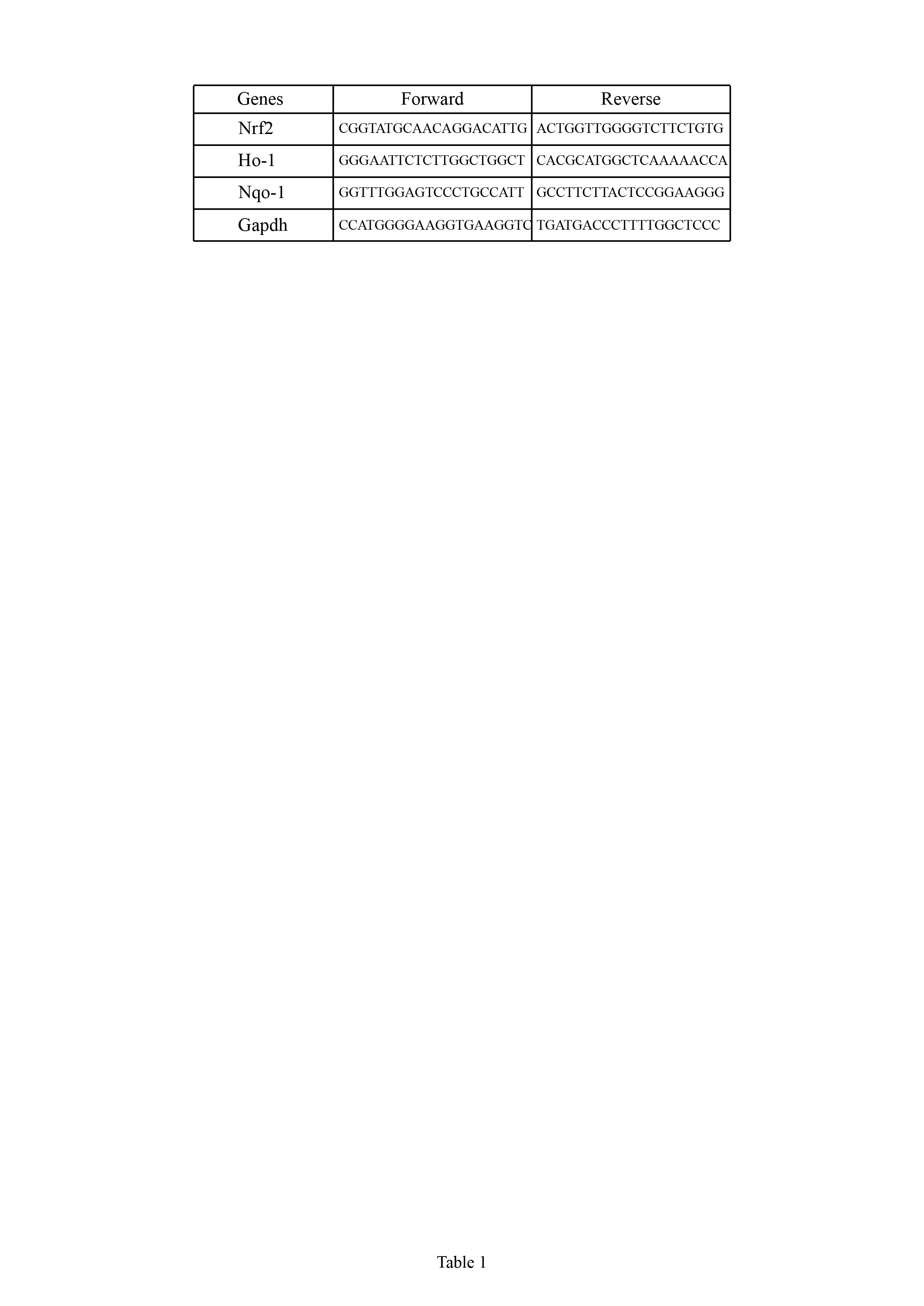

Supplement: Supplementary file 3 [file Image4.JPEG]

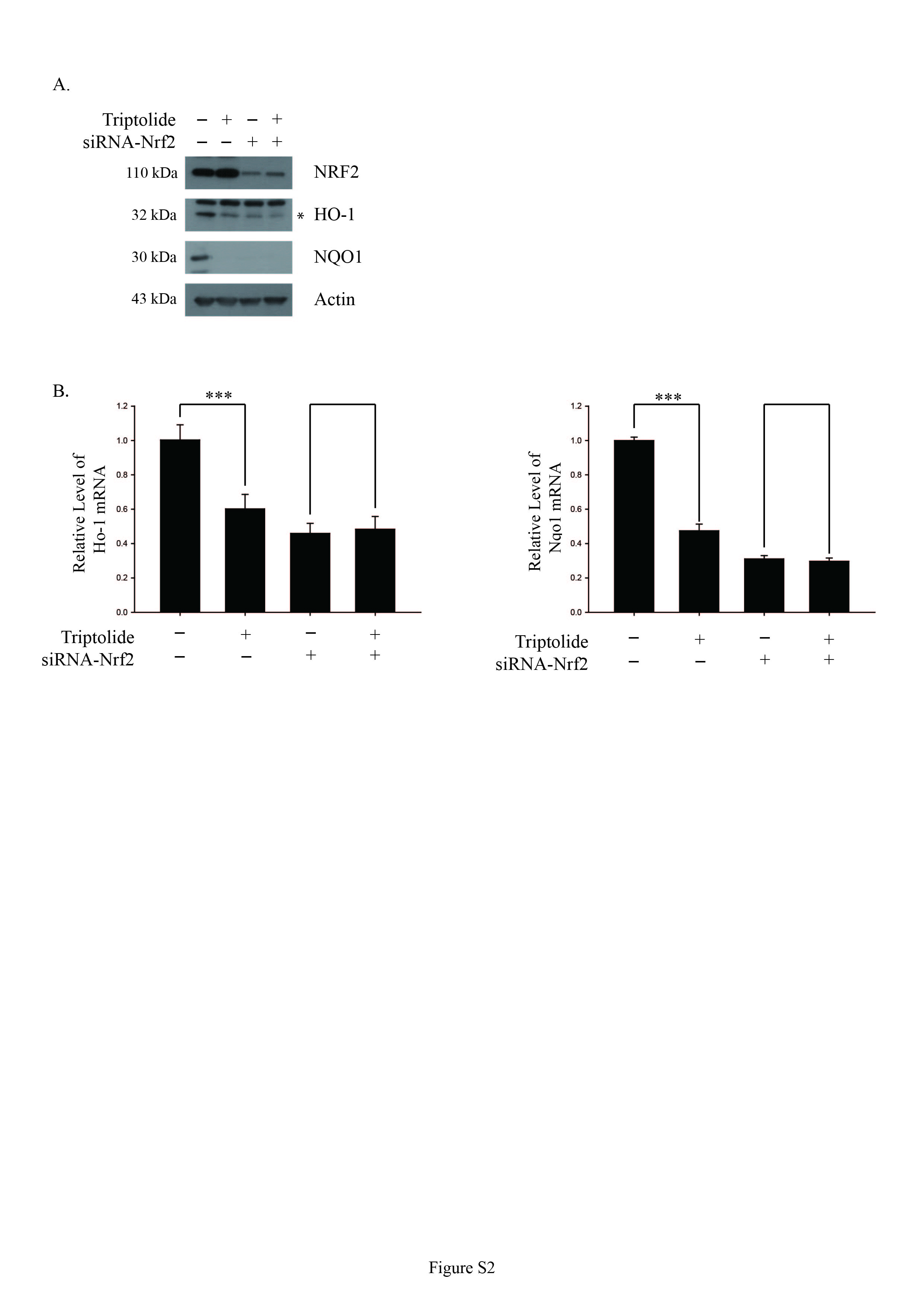

Supplement: Supplementary file 4 [file Image2.JPEG]
